# Aging Exacerbates Ischemia-Reperfusion-Induced Mitochondrial Respiration Impairment in Skeletal Muscle

**DOI:** 10.3390/antiox8060168

**Published:** 2019-06-08

**Authors:** Stéphanie Paradis, Anne-Laure Charles, Isabelle Georg, Fabienne Goupilleau, Alain Meyer, Michel Kindo, Gilles Laverny, Daniel Metzger, Bernard Geny

**Affiliations:** 1 Fédération de Médecine Translationnelle de Strasbourg, Faculté de Médecine, Institut de Physiologie, Equipe d’Accueil EA3072 “Mitochondrie, Stress Oxydant et Protection Musculaire”, Université de Strasbourg, 67000 Strasbourg, France; stephanie.paradis@wanadoo.fr (S.P.); anne.laure.charles@unistra.fr (A.-L.C.); isabelle.georg@unistra.fr (I.G.); goupilleau@unistra.fr (F.G.); alain.meyer1@chru-strasbourg.fr (A.M.); michel.kindo@chru-strasbourg.fr (M.K.); 2 Service de Physiologie et d’Explorations Fonctionnelles, Pôle de Pathologie Thoracique, Nouvel Hôpital Civil, CHRU de Strasbourg, 67000 Strasbourg, France; 3 Service de Chirurgie Cardiaque, Pôle de Pathologie Cardiaque, Nouvel Hôpital Civil, CHRU de Strasbourg, 67000 Strasbourg, France; 4 Department of Functional Genomics and Cancer, Institut de Génétique et de Biologie Moléculaire et Cellulaire (IGBMC), INSERM U1258, CNRS UMR 7104, Université de Strasbourg, 67404 Illkirch, France; laverny@igbmc.fr (G.L.); metzger@igbmc.fr (D.M.)

**Keywords:** peripheral arterial disease, ischemia-reperfusion, skeletal muscle, mitochondria, respiration, calcium, oxidative stress, reactive oxygen species, aging

## Abstract

Cycles of ischemia-reperfusion (IR) that occur during peripheral arterial disease (PAD) are associated with significant morbi-mortality, and aging is an irreversible risk factor of PAD. However, the effects of advanced age on IR-induced skeletal muscle mitochondrial dysfunction are not well known. Young and aged mice were therefore submitted to hindlimb IR (2 h ischemia followed by 2 h reperfusion). Skeletal muscle mitochondrial respiration, calcium retention capacity (CRC) and reactive oxygen species (ROS) production were determined using high resolution respirometry, spectrofluorometry and electronic paramagnetic resonance. IR-induced impairment in mitochondrial respiration was enhanced in old animals (V_ADP_; from 33.0 ± 2.4 to 18.4 ± 3.8 and 32.8 ± 1.3 to 5.9 ± 2.7 pmol/s/mg wet weight; −44.2 ± 11.4% vs. −82.0 ± 8.1%, in young and aged mice, respectively). Baseline CRC was lower in old animals and IR similarly decreased the CRC in both groups (from 11.8 ± 0.9 to 4.6 ± 0.9 and 5.5 ± 0.9 to 2.1 ± 0.3 µmol/mg dry weight; −60.9 ± 7.3 and −60.9 ± 4.6%, in young and aged mice, respectively). Further, IR-induced ROS production tended to be higher in aged mice. In conclusion, aging exacerbated the deleterious effects of IR on skeletal muscle mitochondrial respiration, potentially in relation to an increased oxidative stress.

## 1. Introduction

Ischemia-reperfusion (IR) cycles that occur during peripheral arterial disease (PAD) induce skeletal muscle injury resulting in lifestyle-limiting disability, including exercise intolerance and in the most severe cases, limb amputation. PAD is also often associated with other cardiovascular diseases including myocardial infarction, stroke and cognitive dysfunctions. With more than 200 million people diagnosed in the world, PAD is a public health issue. PAD prevalence is high in aged people and although individuals over the age of 70 present the highest risk, revascularization in nonagenarian patients appeared effective as long as the patient was not fully dependent [1,2,3,4,5,6]. However, improved therapeutic options based on PAD pathophysiology are still needed.

PAD pathophysiology clearly demonstrated a major role for skeletal muscle mitochondrial dysfunction and oxidative stress, which promote cell damage and death. Thus, oxidative phosphorylation capacity is reduced, hampering adenosine triphosphate (ATP) generation [7,8,9,10,11,12,13]. Interestingly, further underlining the importance of mitochondria, mitochondrial content in skeletal muscle demonstrated to be a good predictor of the mortality rate during PAD in humans [14]. Thus, improving our knowledge of the mitochondrial implication during IR, particularly in aging individuals, appears mandatory. Indeed, aging is also known to induce sarcopenia (i.e., skeletal muscle weakness and atrophy), associated with decreased number and size of fibers, alteration of the excitation-contraction coupling, slow-to-fast switch of fiber type, denervation and decrease of regeneration capacity [15,16,17], however, few data have been reported on the effects of aging on IR injury in skeletal muscle [18,19,20,21,22]. We previously observed in aged rats that mitochondrial respiration and calcium retention capacity (CRC) were impaired after IR in the gastrocnemius muscles, and that aging likely reduced the potential beneficial effect of cyclosporine, considered as a mitochondrial permeability transition pore (mPTP) opening inhibitor [23]. However, the effect of aging was not directly compared in old and young rats and such comparisons still need to be performed in several species.

The aim of this study was therefore to investigate whether old age enhances IR-induced skeletal muscle mitochondrial alterations, analyzing both mitochondrial oxidative and calcium retention capacities, together with reactive oxygen species (ROS) production in young and aged mice submitted to unilateral hindlimb IR.

## 2. Materials and Methods

### 2.1. Animals

Experiments were performed on young (23 ± 1 weeks old) and aged (84 ± 1 weeks old) male C57Bl6J mice. The mice were housed in a neutral temperature environment (22 ± 2 °C) on a 12 h light-dark cycle. Standard food and water were available ad libitum. All experiments were performed in agreement with the French laws for animal use and care and the guidelines from directive 2010/63/EU. The experiments were approved by the local ethics committee and the French Research Minister (agreement number 2018041811246867).

### 2.2. Experimental Procedure

Mice were anesthetized in a hermetic cage, ventilated with gas mixture of 4% isoflurane (Aerrane, CSP, Cournon, France) and oxygen. They were placed on heating blankets (Homeothermic blanket control unit, MINERVE, Harvard Apparatus^®^, Esternay, France) to maintain animal body temperature near 37 °C. Spontaneous ventilation was allowed through the oxygen delivering mask. Depth of anesthesia was checked by tail clamp before more noxious stimuli, and controlled by adapting isoflurane concentrations.

**Figure 1:**
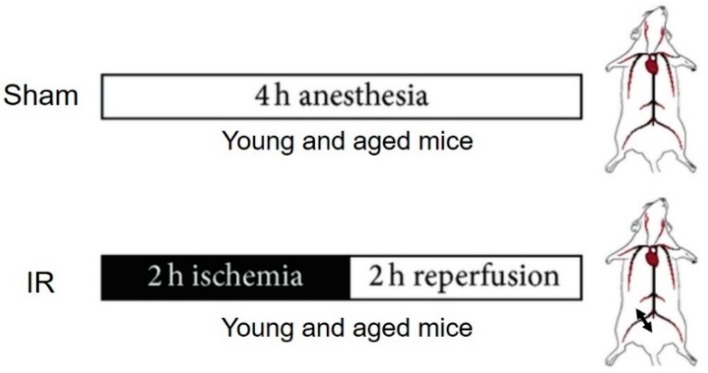
Experimental design of the ischemia-reperfusion (IR) procedure.

Mice were divided into four groups (Figure 1). Young and aged sham groups (Y-Sham (*n* = 6) and A-Sham (*n* = 5)), were submitted to 4 h anesthesia. Two other young and aged groups were submitted to 2 h ischemia with a tourniquet placed around the right hindlimb, at the level of the groin, followed by 2 h reperfusion (Y-IR (*n* = 7), A-IR (*n* = 7), respectively). Ischemia was ascertained by visual observation of cyanosis of the limb and mice were sacrificed at the end of the experiment [13].

### 2.3. Processing of Skeletal Muscle Tissue

The superficial gastrocnemius muscles (i.e., glycolytic), characterized by a greater susceptibility to IR-induced damage [24], were harvested and immediately placed in a Krebs-HEPES buffer (NaCl 99 mM, KCl 4.69 mM, CaCl_2_ 2.5 mM, MgSO_4_ 1.2 mM, NaHCO_3_ 25 mM, KH_2_PO_4_ 1.04 mM, D(+) glucose 5.6 mM, Na-HEPES 20 mM, pH 7.4 at 4 °C) allowing preservation of muscle fibers in a physiological environment at 4 °C. Samples were either gently dissected on ice under a dissecting microscope for mitochondrial respiration and CRC measurements or cut into 1–2 mm^3^ slices for assessment of ROS production.

### 2.4. Permeabilization of Skeletal Muscle Fibers

After dissection, muscle fibers were permeabilized by incubation under stirring for 30 min at 4 °C in a buffer S (CaK_2_EGTA 2.77 mM, K_2_EGTA 7.23 mM, Na_2_ATP 6.04 mM, MgCl_2_ 6.56 mM, taurine 20 mM, Na_2_Phosphocreatine 12.3 mM, imidazole 20 mM, dithiothreitol 0.5 mM, K-methane sulfonate 50 mM, pH 7.0 at 4 °C) with saponin (50 µg/mL). Then fibers were rinsed with agitation for 10 min at 4 °C in the buffer S. This technique allowed mitochondrial morphology to be kept and the functional cellular environment to be preserved [25,26].

### 2.5. Study of Mitochondrial Respiration by Oxymetry

Measuring oxygen consumption in permeabilized fibers was performed using a Clark electrode in a thermostated oxygraphic chamber at 37 °C with continuous stirring (Oxygraph-2k, Oroboros instruments, Innsbruck, Austria). Briefly, fibers (3–4 mg wet weight) were incubated twice for 5 min with agitation at 4 °C in a buffer R+ (CaK_2_EGTA 2.77 mM, K_2_EGTA 7.23 mM, MgCl_2_ 1.38 mM, imidazole 20 mM, taurine 20 mM, dithiothreitol 0.5 mM, K-methane sulfonate 90 mM, Na-methane sulfonate 10 mM, glutamate 5 mM, malate 2 mM, K_2_HPO_4_ 3 mM, and bovin serum albumin 2 mg/mL, pH 7.0 at 22.1 °C). Then fibers were placed in 2 mL of buffer R+ in the oxygraphic chamber and basal oxygen consumption (V_0_) was measured. Subsequently, several substrates were added in order to specifically activate different complexes of the mitochondrial electron transport chain (ETC). Addition of a saturating amount of adenosine diphosphate (ADP) (2 mM) allowed measuring complex I, III and IV activity (V_ADP_). Then, a saturating amount of succinate (25 mM) allowed studying complex I, II, III and IV activity (V_Succ_). Finally, addition of N,N,N′,N′-tetramethyl-p-phenylenediamine (TMPD; 0.5 mM) and ascorbate (0.5 mM) specifically activated complex IV (V_TMPD/Asc_). The ratio V_ADP_/V_0_ was calculated to evaluate the degree of coupling between oxidation and phosphorylation. Results were expressed as pmol/s/mg wet weight.

### 2.6. Calcium Retention Capacity Measurements in Ghost Fibers

Opening of the mPTP was assessed by monitoring CRC of skeletal muscle mitochondria under energized conditions, as previously described [13]. Briefly, permeabilized fibers (5–6 mg wet weight) were incubated for 30 min under stirring at 4 °C in buffer R+ supplemented with KCl (800 mM) to extract myosin, block the calcium uptake by the sarcoplasmic reticulum, and thus allow calcium uptake only by mitochondria. Then fibers were washed 3 times 10 min in CRC buffer (Tris-Base 20 mM, saccharose 150 mM, KCl 50 mM, KH_2_PO_4_ 2 mM, succinate 5 mM, pH 7.4 at 23 °C) containing ethylene glycol-bis(β-aminoethyl ether)-N,N,N′,N′-tetraacetic acid (EGTA) (5 μM) and bovine serum albumin (2 mg/mL).

Permeabilized ghost fibers were incubated at 24 °C in a quartz tank with continuous stirring in 1 mL of CRC buffer containing calcium green-5N fluorescent probe (5 µM; excitation 500 nm; emission 530 nm). The reaction was started by the addition of a calcium pulse (20 mM), following by calcium pulses every 5 min until it was necessary. After each pulse, a peak of extramitochondrial calcium was recorded and a rapid uptake by the mitochondria was observed, resulting in a decrease in extramitochondrial calcium concentration to near basal level. When mitochondria reached the maximal calcium loading threshold, opening of mPTP happens and mitochondrial calcium is released, resulting in an abrupt increase in extramitochondrial calcium concentration. The amount of calcium necessary to trigger mPTP opening was calculated from a standard curve relating calcium concentrations to the fluorescence of calcium green-5N. At the end of the experiment, muscle fibers were gathered, dehydrated at 150 °C for 15 min, and weighted. Results were expressed as µmol/mg dry weight.

### 2.7. Measurement of Intramuscular Reactive Oxygen Species Production by Electron Paramagnetic Resonance Spectroscopy

The electron paramagnetic resonance spectroscopy is one of the best techniques used to detect the “instantaneous” presence of free radical species in the samples, by oxidation from superoxide anion (O_2_^·^) and other ROS of a spin probe 1-hydroxy-3-methoxycarbonyl-2, 2, 5, 5-tetramethyl-pyrrolidine (CMH; oxidized form CM^·^, Noxygen^®^, Elzach, Germany). Muscle fragments were incubated for 30 min in Krebs-HEPES buffer containing deferoxamine (25 μM), diethyldithiocarbamate (5 μM) and CMH (200 µM) in a thermoregulated incubator at 37 °C under gas mix (O_2_: 2.7%, N_2_: 97.8%) and controlled pressure (20 mmHg; Gas Treatment Chamber BIO-V and Temperature & Gas Controller BIO-III, Noxygen^®^, Elzach, Germany). Then the reaction was stopped on ice and all experiment measures of oxidized probe concentration were performed at 15 °C in disposable capillary tubes from 40 µL of supernatant, using the e-scan spectrometer (Bruker Win-EPR^®^, Elzach, Germany), as described previously. Detection of ROS was conducted under the following settings, center field 3461.144 g, microwave power 21.85 mW, modulation amplitude 2.40 g, sweep time 5.24 s (10 scans), sweep width 60 g, number of lag curve points 1. The signal amplitude is calculated and the concentration of CM^·^ is determined from standard calibration curve of CM. At the end of the experiment, muscle fragments were gathered, dehydrated at 150 °C for 15 min, and weighted. Results were expressed in μmol/min/mg dry weight.

### 2.8. Statistical Analysis

Values are represented by mean ± SEM. The statistical analysis was performed using a two-way ANOVA test, followed by the Tukey post-test. A *p*-value less than 0.05 was considered significant.

## 3. Results

### 3.1. Ischemia-Reperfusion-Induced Mitochondrial Respiration Dysfunction was Exacerbated by Aging

**Figure 2:**
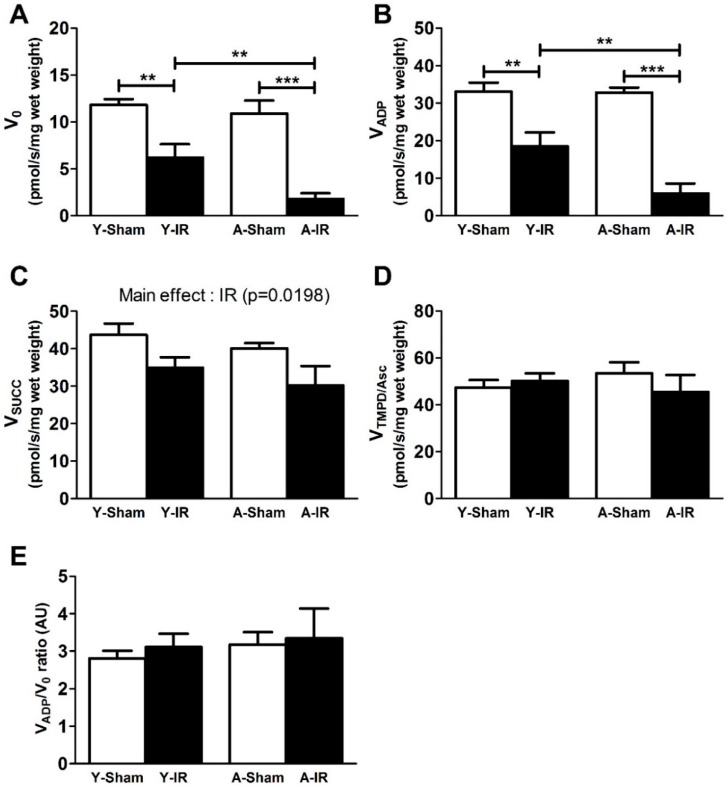
Aging exacerbated ischemia-reperfusion-induced mitochondrial respiration dysfunction. Y: Young. A: Aged. White bar: Sham. Black bar: Ischemia-reperfusion (IR). (**A**): Basal oxygen consumption with glutamate and malate (V_0_). (**B**): Complex I, III and IV activity by addition of adenosine diphosphate (ADP) (V_ADP_). (**C**): Complex I, II, III and IV activity by addition of succinate (V_Succ_). (**D**): Complex IV activity by addition of N,N,N′,N′-tetramethyl-p-phenylenediamine (TMPD) and ascorbate (V_TMPD__/Asc_). (**E**): V_ADP_/V_0_ ratio. Results are presented as mean ± SEM. **: *p* < 0.01 and ***: *p* < 0.001.

The activity of the complexes of the mitochondrial ETC was measured in sham and IR limbs of young and aged mice, as shown in Figure 2. In young mice, IR decreased V_0_ (from 11.8 ± 0.6 to 6.2 ± 1.4 pmol/s/mg wet weight, −47.6 ± 12.1%, *p* < 0.01 and V_ADP_ (from 33.0 ± 2.4 to 18.4 ± 3.8 pmol/s/mg wet weight, −44.2 ± 11.4%, *p* < 0.01). Such impairment was enhanced in old mice V_0_ (from 10.9 ± 1.4 to 1.8 ± 0.6 pmol/s/mg wet weight, −83.5 ± 5.4%, *p* < 0.001) and V_ADP_ (from 32.8 ± 1.3 to 5.9 ± 2.7 pmol/s/mg wet weight, −82.0 ± 8.1%, *p* < 0.001) (Figure 2A,B).

Considering the mitochondrial respiration through complex II-linked succinate substrate, V_Succ_ decreased from 43.7 ± 3.0 to 35.0 ± 2.8 and from 40.1 ± 1.5 to 30.2 ± 5.2 pmol/s/mg wet weight, in sham and IR young and aged mice, respectively. A main effect of IR (*p* = 0.0198) was observed, without a specific effect of aging (*p* = 0.2683) (Figure 2C).

No change in mitochondrial respiration was observed when complex IV activity (V_TMPD/Asc_) was activated (Figure 2D, 47.3 ± 3.3 vs. 50.2 ± 3.2 and 53.5 ± 4.7 vs. 45.4 ± 7.3 pmol/s/mg wet weight, in sham vs. IR in young and aged mice, respectively).

The respiratory control capacity (i.e., V_ADP_/V_0_ ratio), representing the degree of coupling between oxidation and phosphorylation, remained unchanged in all groups (Figure 2E, 2.8 ± 0.2 vs. 3.1 ± 0.4 and 3.2 ± 0.3 vs. 3.3 ± 0.8 arbitrary unit, in sham vs. IR in young and aged mice, respectively).

### 3.2. Calcium Retention Capacity

**Figure 3:**
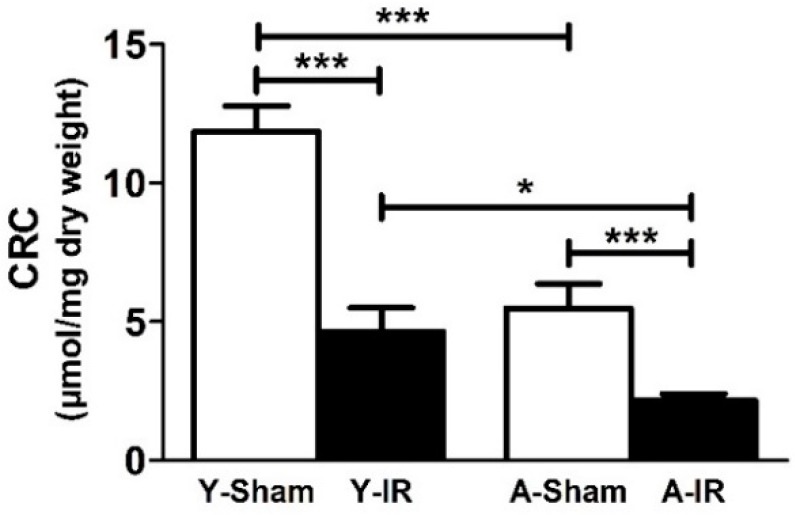
Baseline calcium retention capacity was lower in old mice and ischemia-reperfusion further impaired it in both young and old mice. CRC: Calcium retention capacity. Y: Young. A: Aged. White bar: Sham. Black bar: Ischemia-reperfusion (IR). Results are presented as mean ± SEM. *: *p* < 0.05 and ***: *p* < 0.001.

Figure 3 shows the resistance of mPTP opening in response to the calcium challenge in young and aged mice. Aged sham mice demonstrated a significant decrease in mitochondrial CRC, as compared to young ones (11.8 ± 0.9 vs. 5.5 ± 0.9 µmol/mg dry weight, *p* < 0.001). IR further and similarly reduced the CRC in both groups (−60.9 ± 7.3% vs. −60.9 ± 4.6%, *p* < 0.001 in young and aged mice, respectively).

### 3.3. Reactive Oxygen Species Production

**Figure 4:**
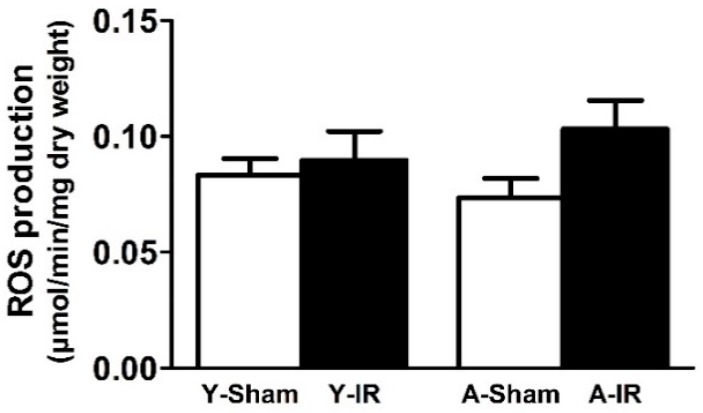
Reactive oxygen species production. ROS: Reactive oxygen species. Y: Young. A: Aged. White bar: Sham. Black bar: Ischemia-reperfusion (IR). Results are presented as mean ± SEM.

IR did not significantly modify ROS production (0.083 ± 0.007 vs. 0.090 ± 0.013 and 0.074 ± 0.008 vs. 0.103 ± 0.012 µmol/min/mg dry weight, in young and aged mice, respectively). However, ROS tended to increase in aged animals (+40.0 ± 16.8%, Figure 4).

## 4. Discussion

The main findings of this study demonstrate that aging exacerbates lower limb IR-induced mitochondrial respiration impairment in skeletal muscle. Such enhanced alteration was observed while ROS production tended to increase in the aged muscle. Baseline CRC was reduced in aged mice, but the further IR-induced impairment in CRC was similar in both groups.

### 4.1. Experimental Design and Baseline Characteristics of Young and Aged Muscle

As few data investigating the effects of age on IR-related deleterious effects on skeletal muscle oxidative capacity were reported, we used a well-known model of acute tourniquet-induced IR inducing mitochondrial respiratory chain alterations [13]. We specifically harvested the gastrocnemius muscle since this glycolytic phenotype has been described to be more sensitive to IR [24,27,28,29], and to present a greater atrophy with aging [30,31,32].

We also selected in vivo markers of mitochondrial functions relevant to sarcopenia associated with aging and to IR injury. Specifically, we examined mitochondrial respiratory chain complex activities, mPTP opening through the mitochondrial sensitivity to calcium overload, and ROS production as these parameters are very often impacted in skeletal muscle. Indeed, IR and aging have been independently implicated in the impairment of mitochondrial respiration, in increased ROS production and oxidative damages, and in mPTP opening changes [7,8,10,13,26,33,34,35,36,37,38,39,40,41,42,43,44,45,46].

In the current study, we did not find baseline (i.e., before IR) difference in mitochondrial respiration and ROS production between young (6 months old) and aged (21 months old) sham mice. These data were similar to others studying mitochondrial respiration [47,48] and oxidative stress production [49]. However, and interestingly, we observed a decrease in mitochondrial CRC with aging. This is consistent with a previous work in glycolytic permeabilized fibers showing a reduction in mitochondrial CRC and time of mPTP opening with aging [31]. Since intracellular calcium concentration increases with aging in skeletal muscle [50], this might jeopardize the aged muscle.

### 4.2. Aging Exacerbated IR-Induced Skeletal Muscle Injuries

In young mice, as expected, although ROS production was not significantly increased, as has been observed previously in a similar setting reporting no ROS production at all [13,21], we observed deleterious effects of IR on both mitochondrial respiration and CRC. While impaired mitochondrial respiration is well-known, there are only several reports demonstrating an impaired mitochondrial CRC in skeletal muscle after lower limb IR [45,51,52,53]. Such increased susceptibility of mPTP opening is thought to favor cell death, either by apoptosis [10,45], or by necrosis [54,55].

Considering aging, few studies specifically investigated a possible enhanced sensitivity of old muscle as compared to young muscles in response to lower limb IR and therefore analyzed concomitantly of both young and aged muscles in such a setting [19,20,21,22]. A recent review confirms the need of research on the effect of aging on IR-induced ROS production [56]. Here, combining the effects of aging and IR on skeletal muscle mitochondrial functions, we demonstrated that aging exacerbated IR-induced mitochondrial ETC dysfunction, mainly affecting complex I activity. Similarly, Miura et al. demonstrated that aging decreased blood flow recovery and angiogenesis after IR, through mechanisms implying damages of mitochondrial DNA, ROS production and muscle mitochondrial dysfunction [22]. Accordingly, previous data reported that IR further altered muscle morphology and force in older rats [19,20].

Interestingly, relatively little is known on the effect of aging on mPTP opening susceptibility after IR in skeletal muscle. We observed that unlike mitochondrial respiration, the IR-induced CRC decrease was not enhanced in old mice. IR-related CRC alterations were nevertheless significant but, they were similar in young and old mice. Whether this might be related to the already decreased baseline CRC value in old muscle or if it is species specific, deserves further studies. Interestingly, even if heart and skeletal muscles differ on some aspects, according to our data, isolated cardiac mitochondria of aged rats showed increased mPTP opening susceptibility after IR [57]. This might be related to a calcium surcharge in the cytoplasm of aged cardiomyocyte [58].

### 4.3. Limitations of the Study

Several limitations deserve to be acknowledged. We did not investigate gender-associated differences that might have modulated both aging and IR-effects on skeletal muscles. This deserves to be studied. Further, although such a preclinical model is well-characterized and exhibits pathophysiological features similar to those observed in patients, this is an experimental study performed in healthy mice without key cardiovascular risk factors such as diabetes, hypertension etc. Thus, although the message of an enhanced susceptibility to IR-damage in aging might be even stronger when taking into account other risk factors, caution should be applied when extrapolating these data to humans.

## 5. Conclusions

The present study provides evidence that aging exacerbates the deleterious effects of IR in skeletal muscle, further impairing mitochondrial respiration. Although not affecting the mitochondrial CRC, already impaired in old mice, such enhanced susceptibility to IR of aged muscles should likely be taken into account when treating older patients suffering from PAD.
